# Phubbing and temperaments among young Lebanese adults: the mediating effect of self-esteem and emotional intelligence

**DOI:** 10.1186/s40359-021-00594-7

**Published:** 2021-05-22

**Authors:** Zeinab Bitar, Souheil Hallit, Wael Khansa, Sahar Obeid

**Affiliations:** 1 Research Department, Psychiatric Hospital of the Cross, P.O. Box 60096, Jall-Eddib, Lebanon; 2 Faculty of Medicine and Medical Sciences, Holy Spirit University of Kaslik (USEK), Jounieh, Lebanon; 3 INSPECT-LB: National Institute of Public Health, Clinical Epidemiology and Toxicology, Beirut, Lebanon; 4 Faculty of Arts and Sciences, Holy Spirit University of Kaslik (USEK), Jounieh, Lebanon

**Keywords:** Phubbing, Temperaments, Self-esteem, Emotional intelligence, Lebanese adults

## Abstract

**Background:**

The rapid increasing rate of mobile and internet users in Lebanon, predisposes us to a high dependency on smartphones, leading to more phubbing. Phubbing has been found associated with many psychological factors. Thus, the main objectives of this study was (1) to evaluate the association between phubbing and temperaments, and (2) assess the mediating effect of self-esteem and emotional intelligence in the association between phubbing and temperaments among a sample of Lebanese adults.

**Methods:**

A cross-sectional study, carried out between August and September 2020, enrolled 461 participants aged between 18 and 29 years old. Participants were recruited from all districts/governorates of Lebanon (Beirut, Mount Lebanon, North Lebanon, South Lebanon, and Bekaa) using the snowball technique. The Generic Scale of Phubbing, Rosenberg Self‐Esteem Scale, Schutte Self Report Emotional Intelligence Test and TEMPS-M were used to assess phubbing, self-esteem, emotional intelligence and temperaments respectively.

**Results:**

Our results showed that higher depressive temperament (B = 1.21) was significantly associated with more phubbing, whereas higher self-esteem (B = − 0.32) was significantly associated with less phubbing. Regarding the mediating effect, self-esteem partially mediated the association between depressive temperament and phubbing (21.02%), whereas emotional intelligence had no mediating effect on the association between temperaments and phubbing.

**Conclusion:**

A strong correlation between phubbing and temperaments has been found in our study with a partial mediating effect of self-esteem in this association. Our findings might be a first step for raising awareness to develop the etiquette of using smartphones by providing media education to families, and good media usage habits.

## Background

Phubbing is a word that has been recently used to describe people snubbing on phones [1]. This has emerged alongside the major advancement and development of mobile technologies and the effortless accessibility to the internet, which increased the use and preference for mobile devices among people [2].

The development done in the mobile industry made it easy for the normal user to do many tasks that were at one time labeled as desk tasks, as you can book flight tickets with one click when commuting on public transport or during work coffee/lunch breaks [3, 4]. These features developed a never seen before dependency on smartphones, where the phone has become part of the person’s daily life [4].

As of 2020, mobile users have reached a number of 5.2 billion people, with this number expected to increase to 17 billion people by 2024. This includes 4.6 billion internet users (90% accounting for mobile users) and a number of 4.1 billion social media users [5]. All these numbers are also marked by an increase in global digital growth, and increased screen time, reaching more than 10 billion hours per day of social media used worldwide, equivalent to more than 1 million years of human existence [5]. These numbers also come in parallel with the coronavirus pandemic (COVID-19) during which social distancing was its main character [6]. During this pandemic, multiple countries went through long period of lockdowns, distanced learning experience, and online conferences, all of which increasing internet usage [7–9].

All this resulted in a phenomena of using the smartphone even when a person is engaged in face-to-face encounters, a behavior described as “Phubbing” [10]. The phubber is the person engaging with his mobile during a person-to-person interaction, while the person/group being phubbed is/are called phubbee(s) [1]. When phubbing occurs, people report a feeling of distractibility, decreased level of joy regarding the person-to-person communication [11], less quality of communication [12] and a decreased level of interpersonal trust [13]. While many agree that phubbing is annoying and not acceptable, this behavior has seen an increased number of people engaging in it. Previous findings conducted in the United States showed that 46.3% of respondents said their partners phubbed them, whereas 22.6% said it caused them relationship issues [13].

Nevertheless, phubbing is considered an impactful behavior at many levels. Literature mentions an increased feeling of jealousy [14], decreased intimacy within partners [15], decreased relationship satisfaction [16], and increased depressive symptoms among people in relationships [17] [18]. It was also associated with an increase in social withdraw [19, 20] and mental health problems [21]. Furthermore, being phubbed was correlated with decreased trust toward the phubber [22], and a decrease in perception of communication quality [23].

How individuals perceive the behavior of phubbing and the reason behind it were also of big interest in multiple studies. Literature mentions many behaviors as predictors of phubbing such as internet addiction, decreased self-control, fear of missing out [1, 24, 25], social media addictions [24, 26], neuroticism, social anxiety and anxiety traits [27].

Risk factors that predispose to internet addiction (IA) should be detected early to decrease the consequences of IA; temperament is one of those risk factors [28]. Temperament is defined as “temporally stable biological core of personality”. It presents the patterns of emotions, moods, activity level and sensory responses that characterize an individual. It is shaped by societal norms and values, and is associated with specific brain systems that regulate negative emotions and behaviors [29]. The traditional psychopathological model commonly includes four affective temperamental types (e.g. depressive, hyperthymic, cyclothymic, and irritable), with the anxious temperament being added later on to it [30]. The cyclothymic temperament is characterized by chronic cycling between mood polarities and unstable self-esteem and energy; the hyperthymic temperament by increased energy and optimism [31], the irritable temperament by irritable and angry behavior; the anxious temperament by a tendency to worry; and depressive temperament by low levels of energy, introversion and worrying [30]. Previous findings showed a strong association between phubbing and these five temperaments [32].

Also, self-esteem has been previously presented as an antecedent for phubbing [33]. Self-esteem defines a person’s overall sense of his or her worth; it is a measure of how much a person “values, approves of, appreciates, prizes, or likes him or herself” [34]. Some correlation existed between internet addiction and self-esteem especially among adolescents and young adults [33, 35, 36].

Emotional intelligence is defined as the capability of individuals to recognize their own and others’ emotions, differentiate between various feelings and label them appropriately, use emotional information to guide thinking and behavior, and adjust emotions to adapt to environments [37]. A reverse relationship has been found between emotional intelligence and internet addiction [38, 39]. Also, low emotional intelligence is a predictor of addiction-related behavior such as internet use, gambling and video game playing [25].

Given the novelty of this topic and the emergence of the COVID-19 pandemic, phubbing has become a habit seen in every single household, community and society, raising many questions around it. These questions pushed researchers to study a variety of important variables that might be correlated with this habit. In Lebanon, there is no study done evaluating the rise of this phenomenon to this day. To add, the country was suffering from the emergence of the COVID-19 with the lockdown imposed on the Lebanese people. The country is also going through a severe economic crisis in which unemployment rate has reached around 30% mark estimated by a Lebanese consulting firm [40].

The estimated number of internet users in Lebanon as of January, 2021, was 5.31 million users, with reported penetration rate of 78.2% [41]. Previous findings showed that internet addiction prevalence rate is 16.8% among university students in Lebanon [42] and varies between 25.2% [43] and 43.6% [36]among Lebanese adolescents. In many studies, a strong correlation has been found between internet addiction and higher stress, anxiety, and depression [32, 42] on one hand, and lower self-esteem and emotional intelligence [33, 44] on another hand. Therefore, since the correlation between temperament, self-esteem and emotional intelligence to phubbing has not been studied thoroughly, the main objectives of this study were (1) to evaluate the association between phubbing and temperaments, and (2) assess the mediating effect of self-esteem and emotional intelligence in this association among a sample of Lebanese adults.

## Methods

### General study design

A cross-sectional study was carried out between August and September 2020, during the lockdown period imposed by the government for the COVID-19 pandemic, which coincides with the summer vacation for most Lebanese.

#### Participants

We used a sample of community-dwelling participants aged 18–29 years. We chose this age range since young adults are more inclined toward using mobile phones [45]. All participants in this age group, who had a mobile phone, were eligible to participate. Excluded were those who refused to fill out the questionnaire.

#### Procedure

Due to the restrictions on gatherings and the non-practical and risky side of face-to-face interviews, we used an anonymous self-administered questionnaire developed on Google Forms. The link was shared among participants using the WhatsApp application and sent to people from all districts/governorates of Lebanon (Beirut, Mount Lebanon, North Lebanon, South Lebanon, and Bekaa) using the snowball technique. Participants were asked to fill the survey online and send the link to other smartphone users too, which explains the snowball sampling technique used.

### Minimal sample size calculation

According to the G-power software, and based on an effect size f2 = 2%, an alpha error of 5%, a power of 80%, and taking into consideration 10 factors to be entered in the multivariable analysis (multiple regression), the results showed that a minimal number of 395 was needed.

### Translation procedure

The scales were forward and back-translated. Forward translation (English–Arabic) was performed by one psychologist, whereas the back translation from Arabic to English was performed by another psychologist. Minor discrepancies were solved by consensus.

### Questionnaire and variables

The self-administered questionnaire was in Arabic and had closed-ended questions; it required approximately 25–30 min to be completed. The first part clarified socio-demographic characteristics: age, gender, marital status, work status, educational level, and household crowding index. The latter was calculated by dividing the number of persons in the house by the number of rooms in it (excluding the bathrooms and kitchen); higher household crowding index reflects lower socioeconomic status [46]. The second part of the questionnaire included the following scales:

### Generic scale of phubbing (GSP)

The Generic Scale of Phubbing (GSP) scale was used to measure the unique behavior of phubbing in social interaction [23]. The GSP scale revealed good construct, criterion, discriminant and convergent validities, as well as internal consistency and test–retest reliabilities [23]. This scale consists of fifteen items that are scored on a seven-point Likert scale (0 = never and 7 = always), with higher scores indicating more phubbing. In this study, the Cronbach’s alpha value was 0.929.

### The temperament evaluation in Memphis, Pisa and San Diego scale (TEMPS-M)

In order to assess affective temperaments, the Temperament Evaluation in Memphis, Pisa and San Diego (TEMPS) scale was originally developed in forms of interview (TEMPS-I) [31] or of self-administered questionnaire (TEMPS-A) [30]. Based on TEMPS-A, Erfurth et al.[47] developed the TEMPS-M, a shorter version (35 items), with the items scoring being the main change from the original version. TEMPS-M is composed of 35 self-rating items that can be assigned to 5 subscales: depressive, cyclothymic, hyperthymic, irritable, and anxious. All responses are provided on a 5-point Likert scale ranging from 1 (not at all) to 5 (very much). Subscale scores range from 5 to 35, with higher scores denoting higher expressions of the respective temperament. In this study, the Cronbach’s alpha values for the subscales were as follows: depressive (0.809), cyclothymic (0.898), hyperthymic (0.818), irritable (0.808), and anxious (0.856).

### Rosenberg self‐esteem scale (RSES)

The Rosenberg Self-Esteem Scale (RSES) [48] is a 10-item scale that reflects self-worth by focusing on both positive and negative feelings people have about themselves. Responses were scored from 1 (strongly disagree) to 4 (strongly agree), where higher scores reflect a better self-esteem (Cronbach’s alpha in this study = 0.837).

### Schutte self report emotional intelligence test (SSEIT)

The Schutte Self-Report Emotional Intelligence Test (SSEIT) is a method of measuring general Emotional Intelligence (EI), using four sub-scales: emotion perception, utilizing emotions, managing self-relevant emotions, and managing others’ emotions [49]. The SSEIT is composed of 33 items, scored on a five-point Likert scale (1 = strongly disagree to 5 = strongly agree), with higher scores reflecting higher emotional intelligence [49]. In this study, the Cronbach’s alpha for this scale was 0.967.

### Statistical analysis

The SPSS software version 23 was used to conduct data analysis. The normality of distribution of the phubbing score was confirmed via a calculation of the skewness and kurtosis; values for asymmetry and kurtosis between −2 and + 2 are considered acceptable in order to prove normal univariate distribution [50]. These conditions consolidate the assumptions of normality in samples larger than 300 [51]. The Student t and ANOVA tests were used to compare two and three or more means respectively, whereas the Pearson correlation test was used to correlate two continuous variables. A forward linear regression was conducted to check for correlates associated with phubbing. Cronbach’s alpha values were recorded for reliability analysis of all scales and subscales.

### Mediation analysis

**Fig. 1:**
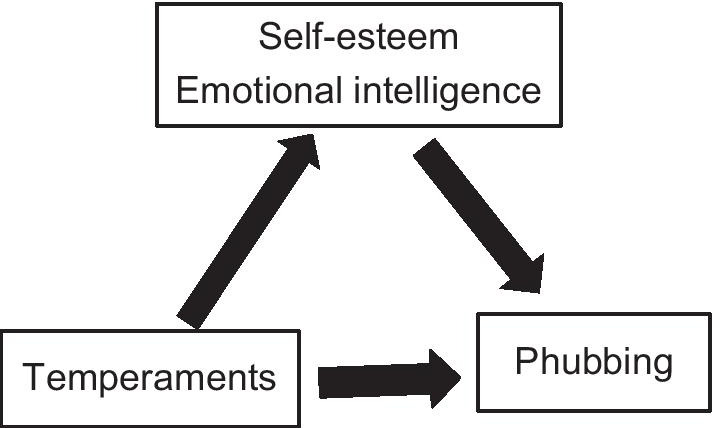
Effect of temperaments on phubbing, mediated by self-esteem/emotional intelligence

The PROCESS SPSS Macro version 3.4, model four was used to calculate three pathways (Fig. 1). Pathway A determined the regression coefficient for the association of temperaments and self-esteem/emotional intelligence, Pathway B examined the association between self-esteem/emotional intelligence and phubbing, independent of the temperaments, and Pathway C’ estimated the total and direct effect of temperaments on phubbing. Pathway AB calculated the indirect intervention effects. To test the significance of the indirect effect, the macro generated bias-corrected bootstrapped 95% confidence intervals (CI) should not include zero. In the linear regression and mediation models, included covariates corresponded to those that showed a *p* < 0.2 in the bivariate analysis. Nagelkerke R^2^ values were also calculated for all models to check how much independent variables would explain the dependent one. Significance was set at a *p* < 0.05.

## Results

A total of 461 persons accepted to participate in this study. The mean age of the participants was 22.25 ± 2.87 years, with 70.9% females. The majority of the participants was single (91.3%) and had a university education level (94.4%). The mean household crowding index was 1.08 ± 0.61 (Table 1). In addition, the means and standard deviations of the scales were as follows: phubbing (41.36 ± 16.94; median = 38), depressive temperament (16.13 ± 5.54), cyclothymic temperament (18.30 ± 6.94), hyperthymic temperament (20.69 ± 5.94), irritable temperament (18.38 ± 5.67), anxious temperament (17.32 ± 6.49), self-esteem (28.48 ± 5.36) and emotional intelligence (112.03 ± 26.02).

**Table 1:** Sociodemographic characteristics of the study sample (N = 461)

	Frequency (%)
Gender	
Male	134 (29.1%)
Female	327 (70.9%)
Marital status	
Single/widowed/divorced	421 (91.3%)
Married	40 (8.7%)
Education level	
School education	26 (5.6%)
University education	435 (94.4%)
	Mean ± SD
Age (in years)	22.25 ± 2.87
Household crowding index	1.08 ± 0.61

### Bivariate analysis

A higher mean generic scale of phubbing was significantly found in single participants compared to married ones (41.85 vs 36.15, *p* = 0.032). Furthermore, higher depressive (r = 0.477), cyclothymic (r = 0.451), hyperthymic (r = 0.158), irritable (r = 0.324), and anxious (r = 0.382) temperaments were significantly associated with more phubbing, whereas higher self-esteem (r = −0.234) and emotional intelligence (r = −0.103) were significantly associated with less phubbing (Table 2).

**Table 2:** Bivariate analysis taking the Generic scale of Phubbing as the dependent variable

	Generic scale of Phubbing	*P* value
	Mean ± SD	
Gender		
Male	40.13 ± 16.36	0.382
Female	41.86 ± 17.16	
Marital status		
Single	41.85 ± 17.03	**0.032**
Married	36.15 ± 15.14	
Education level		
School education	41.38 ± 16.38	0.853
University education	41.36 ± 16.98	
	Correlation coefficient	
Depressive temperament	0.477	** < 0.001**
Cyclothymic temperament	0.451	** < 0.001**
Hyperthymic temperament	0.158	**0.001**
Irritable temperament	0.324	** < 0.001**
Anxious temperament	0.382	** < 0.001**
Emotional intelligence	− 0.103	**0.026**
Self-esteem scale	− 0.234	** < 0.001**

Numbers in bold indicate significant p-values

### Multivariable analysis

The results of the linear regression, taking the phubbing score as the dependent variable, showed that higher depressive temperament (B = 1.21) was significantly associated with more phubbing, whereas higher self-esteem (B = −0.32) was significantly associated with less phubbing (Table 3).

**Table 3:** Multivariable analysis

Model 1: Linear regression taking the Generic scale of Phubbing as the dependent variable					
Variable	Unstandardized Beta	Standardized Beta	*p*	95% Confidence Interval	
Depressive temperament	1.21	0.39	< 0.001	0.93	1.48
Self-esteem	− 0.32	− 0.10	0.026	− 0.61	− 0.04

Variables entered in the models: marital status, depressive temperament, cyclothymic temperament, hyperthymic temperament, irritable temperament, anxious temperament, emotional intelligence and self-esteem scale

Adjusted R^2^ = 0.198, *p* < 0.001

Numbers in bold indicate significant *p* values

### Mediation analysis

The results of a first mediation analysis, taking self-esteem as a mediating variable, are summarized in Table 4. Higher depressive temperament was significantly associated with lower self-esteem (B = −0.44, 95% BCa CI [− 0.56, −0.32], t = −7.06, *p* < 0.001 (R^2^ = 0.209) and more phubbing, even with self-esteem in the model (B = 0.75, 95% BCa CI [0.26, 1.23], t = 3.03, *p* = 0.002); higher self-esteem was significantly associated with less phubbing (B = −0.35, 95% BCa CI [− 0.70, −0.01], t =  − 2.02, *p* = 0.043) (R^2^ = 0.205). When self-esteem was not in the model, higher depressive temperament was significantly associated with more phubbing (B = 0.90, 95% BCa CI [0.44, 1.36], t = 3.85, *p* < 0.001 (R^2^ = 0.239). Self-esteem partially mediated the association between depressive temperament and phubbing by 21.02% (Table 4).

**Table 4:** Mediation analysis: Self-esteem as a mediating variable and each temperament as a dependent variable

	Effect of the temperament on self-esteem			Effect of temperament and self-esteem on phubbing			Effect of temperament on phubbing			Mediating effect of self-esteem
	Beta [95% BCa]	t	*p*	Beta [95% BCa]	t	p	Beta [95% BCa]	t	*p*	
Depressive temperament	− 0.44 [− 0.56 to 0.32]	− 7.06	** < 0.001**	0.75 [0.26–1.23]	3.03	**0.002**	0.90 [0.44–1.36]	3.85	** < 0.001**	21.02%
Self-esteem				− 0.35 [− 0.70 to − 0.01]	− 2.02	**0.043**				
Cyclothymic temperament	− 0.19 [− 0.29 to − 0.09]	− 3.75	** < 0.001**	0.20 [− 0.17 to 0.58]	1.05	0.294	0.27 [− 0.10 to 0.64]	1.42	0.156	–
Self-esteem				− 0.35 [− 0.70 to − 0.01]	− 2.02	**0.043**				
Hyperthymic temperament	0.54 [0.45–0.63]	11.91	** < 0.001**	− 0.08 [− 0.46 to 0.30]	− 0.41	0.685	− 0.27 [− 0.61–0.06]	− 1.59	0.112	–
Self-esteem				− 0.35 [− 0.70 to − 0.01]	− 2.02	**0.043**				
Irritable temperament	− 0.001 [− 0.11–0.10]	− 0.03	0.973	0.13 [− 0.27 to 0.52]	0.63	0.530	0.13 [− 0.27 to 0.52]	0.63	0.529	–
Self-esteem				− 0.35 [− 0.70 to − 0.01]	− 2.02	**0.043**				
Anxious temperament	0.004 [− 0.08–0.09]	0.09	0.928	0.24 [− 0.07 to 0.54]	1.51	0.130	0.23 [− 0.07 to 0.54]	1.50	0.134	–
Self-esteem				− 0.35 [− 0.70 to − 0.01]	− 2.02	**0.043**				

Numbers in bold indicate significant *p* values

A second mediation analysis, taking emotional intelligence as a mediating variable, showed that emotional intelligence did not mediate the association between temperaments and phubbing since EI was not significantly associated with phubbing in all models (B = −0.05; 95% BCa −0.11–0.02; t = −1.37; *p* = 0.171) (data not shown).

## Discussion

This study results showed that higher depressive temperament was significantly associated with more phubbing, whereas higher self-esteem was significantly associated with less phubbing. Self-esteem partially mediated the association between depressive temperament and phubbing, whereas emotional intelligence did not mediate the association between any of the temperaments and phubbing.

Our results showed that higher depressive temperament was significantly associated with more phubbing, in accordance with a previous study [32]. Similar results were found in many previous studies where smartphone addiction and Internet addiction were found associated with higher depression [19, 20, 52]. In fact, many studies has showed a strong association between many temperament characteristics and increased risk of depression [53, 54]. Long-term depression can affect various brain regions [55], and can reduce gray matter volume in the caudate nucleus [56], thus, may affect core, biologically rooted psychological structures such as temperaments [57]. In addition, behavioral addiction such as internet addiction, which has been identified as predictor of “phubbing” [24], present the main features of physical and psychological addictions (i.e. mood variability, tolerance, withdrawal, interpersonal conflict and relapse) [58]. Usually the patient uses substances to change his unwanted temperament status and cope with cognitive impairments, a phenomenon called the “self-medication hypothesis” [59]. This act can be similar for internet addiction, where the patient turns to repetitive efforts to go online, decrease the severity of withdrawal symptoms such as depression [38] and deal with underlying psychological problems; this can explain their increased phone and internet use during higher temperaments [60].

Higher self-esteem was associated with lower levels of phubbing in our study, in line with previous findings [33]. The findings of Blachino and colleagues explain that people evaluate themselves with heavy self-criticism and feel unworthy, therefore, turning to excessive internet use to improve their self-esteem [61]. In addition, our mediation analysis showed a partial mediating effect of self-esteem in the association between phubbing and depressive temperament. Some researchers have argued that self-esteem and depression are essentially within the same construct in adults [62]. Moreover, depression has been already showed to be an important risk factor for depressive temperament [57]. This effect has been explained in previous studies where the fear of missing out –defined as “a pervasive apprehension that others might be having regarding experiences from which one is absent” [63]- was associated with irritability, low self-esteem [64, 65] and more depressive symptoms [66]. The fear of missing out has been found strongly associated with higher problematic internet smartphone and internet use [65, 67], which are important predictors of phubbing [24, 26].

### Limitations and strengths

Many limitations could be raised in our study. First, this is a cross-sectional study and cause-effect relationships are not allowed. Second, participants were predominately single, females, and with a university level of education. Also, some questions might have been over- or underestimated by responders, leading to a possible information bias. A residual confounding bias is possible since not all factors associated with phubbing were included in the questionnaire. Finally, the scales used (Generic Scale of Phubbing, TEMPS-M, Rosenberg Self‐Esteem Scale, Schutte Self Report Emotional Intelligence Test) have not been validated in Lebanon. However, this study highlighted many important findings and is the first, to our knowledge, to assess the association between phubbing, temperaments and the mediating effect of emotional intelligence and self-esteem among Lebanese adults.

### Conclusion and Implications for further studies

Our study showed a correlation between phubbing and depressive temperament, with this association being mediated by self-esteem. This study may contribute to the assessment of phubbing and interventions to deal with it, improve the appropriate use of smartphones and develop good media usage habits. Given the limitations of this study, our suggestion is to conduct further studies that will make it possible to look more closely into the relationships between phubbing, temperaments and the mediating effect of obsession, loneliness and gender. In addition, the presented study was conducted in Lebanon; research should be extended to other countries in order to check if the associations we obtained are local or could be extended to other countries.

## Data Availability

All data generated or analyzed during this study are not publicly available to maintain the privacy of the individuals’ identities. The dataset supporting the conclusions is available upon request to the corresponding author.

## Abbreviations

GSP: Generic scale of phubbing
TEMPS-M: The temperament Evaluation in Memphis, Pisa and San Diego scale
RSES: Rosenberg self‐esteem scale
SSEIT: Schutte self report emotional intelligence test
COVID-19: Coronavirus disease
